# French psychiatrists’ concerns about assisted death

**DOI:** 10.1192/j.eurpsy.2025.192

**Published:** 2025-08-26

**Authors:** E. Olie

**Affiliations:** Department of Emergency Psychiatry and Acute Care, Lapeyronie Hospital, CHU Montpellier, Montpellier, France

## Abstract

**Abstract:**

A bill on euthanasia and assisted suicide is currently under discussion in France. It proposes that only competent adults suffering from a serious and incurable condition that threatens their life in the medium term, or who are in an advanced or terminal phase, and experiencing unbearable physical or psychological suffering—either refractory to treatment or considered unbearable in the absence of treatment—may request medical assistance in dying (MAiD). However, French psychiatrists have expressed concerns about the bill, as it does not mandate a psychiatric evaluation, despite the high prevalence of mental disorders, including depression, in the general population. These disorders are even more frequent in end-of-life conditions and can significantly impact decision-making capacity and the wish to die. Depression, a common comorbidity in cancer—the leading cause of MAiD requests—affects approximately 15% of cancer patients but is often underdiagnosed and undertreated. The bill also raises concerns regarding its implications for suicide prevention. Some MAiD requests may stem from treatable psychiatric conditions rather than a well-considered end-of-life choice. Furthermore, a proposed obstruction offense could potentially criminalize suicide prevention efforts, complicating the role of mental health professionals. Uniquely, the French bill allows a third party chosen by the patient to administer the lethal substance, a provision not found in any other country. This raises significant ethical and psychological concerns regarding the emotional burden on the designated individual, who may experience distress, guilt, or long-term psychological repercussions from actively participating in assisted dying. Finally, the possibility of future expansion to include psychiatric-only indications, as seen in other countries, remains a critical issue requiring careful ethical and medical scrutiny.

**Disclosure of Interest:**

None Declared

